# Secretory carcinoma: the eastern Canadian experience and literature review

**DOI:** 10.1186/s40463-018-0315-6

**Published:** 2018-11-16

**Authors:** David Forner, Martin Bullock, Daniel Manders, Timothy Wallace, Christopher J. Chin, Liane B. Johnson, Matthew H. Rigby, Jonathan R. Trites, Mark S. Taylor, Robert D. Hart

**Affiliations:** 1 Division of Otolaryngology – Head & Neck Surgery, Department of Surgery, Dalhousie University, 5820 University Ave. 3rd Floor Dickson Bldg, Halifax, NS B3H 2Y9 Canada; 2 Department of Pathology, Dalhousie University, Halifax, NS Canada; 3 Division of Otolaryngology – Head & Neck Surgery, Department of Surgery, Cumberland Regional Health Care Center, Amherst, NS Canada; 4 Division of Otolaryngology – Head & Neck Surgery, Department of Surgery, Saint John Regional Hospital, Saint John, NB Canada

**Keywords:** Secretory carcinoma, Canada, Salivary gland, ETV6-NTRK3 mutation, Diagnostics

## Abstract

**Background:**

Secretory Carcinoma (SC) is a recently described malignancy affecting salivary glands of the head and neck, with a paucity of evidence regarding the natural history, morbidity, and mortality. This study aimed to investigate the current treatment options utilized for SC, as well as its presentation and outcomes.

**Methods:**

This study is a retrospective case series and includes patients diagnosed with SC at four Maritime Canadian institutions. Literature review of patient outcomes following treatment of SC is also included.

**Results:**

Thirteen patients were identified. Parotid was the most common subsite (69%), followed by minor salivary gland (23%) and submandibular gland (8%). All patients were S100 positive and had at least one additional positive confirmatory stain, including mammaglobin, CK7, or vimentin. Two patients had N2b disease. All patients were treated with primary surgery, and four were offered adjuvant radiotherapy. There was one instance of locoregional recurrence, and one of metastasis. Three patients displayed perineural invasion on pathology, and one patient displayed lymphovascular invasion.

**Conclusion:**

Secretory Carcinoma remains understudied regarding its natural history, presentation, and treatment options. This study is the largest single case series in Canada, and highlights the young age and possible aggressiveness of SC. As well, we provide the most comprehensive literature review to date, with a focus on treatment and outcomes for this disease entity.

**Electronic supplementary material:**

The online version of this article (10.1186/s40463-018-0315-6) contains supplementary material, which is available to authorized users.

## Background

Secretory Carcinoma (SC) is a recently described malignancy affecting salivary glands of the head and neck. Originally named Mammary Analogue Secretory Carcinoma (MASC), the World Health Organization has proposed the unifying title of SC [1]. The salivary glands share many similar features to the breast, including an identical ductulo-acinar architecture [2]. Indeed, MASC was identified due to its morphological and immunohistochemical similarity to the breast tumor Secretory Carcinoma. Further investigation has recently revealed that a subset of salivary gland tumors originally identified as Acinic Cell Carcinoma (AciCC) are actually the disease entity now known as SC.

Secretory Carcinoma has been shown to express the translocation mutation t (12;15) (p13;q25), which results in the fusion gene ETV6-NTRK3[2]. It was the lack of this mutation in AciCC and its subsequent positivity in SC that ultimately lead to the discovery of SC as a distinct disease entity [3]. In fact, the ETV6-NTRK3 mutation is specific to SC among salivary gland neoplasms [4].

The most common updated diagnoses where SC was identified were those originally diagnosed as AciCC, cystadenocarcinoma (not otherwise specified; NOS), adenoid cystic carcinoma, and low-grade carcinoma NOS. Of these, AciCC was by far the most common initial diagnosis[5].

The diagnosis of SC originally relied upon identification of the ETV6-NTRK3 fusion gene through fluorescence in-situ hybridization (FISH). As additional data has been added to the SC literature, identification of a common morphological and immunohistochemistry pattern has been identified, including strong positivity for vimentin, S100, and mammaglobin.

As it is now realized that some neoplasms previously identified as AciCC are actually SC, differences in the natural history of the diseases have been investigated. The evidence is lacking in this comparison, as is data regarding morbidity and mortality related to SC. It has been found that SC may present with cervical lymph node metastasis more commonly than AciCC. This may drive a decrease in both disease-free survival and overall survival[6]. As well, SC may more commonly develop local recurrence and distant metastasis [6]. Despite these findings, many authors have considered SC to generally be non-aggressive, analogous to AciCC.

Given the paucity of studies investigating SC treatment options and patient outcomes, we investigated a series of these patients in four Canadian institutions. Our outcomes are compared to a literature review presented here, the first to focus on secretory carcinoma outcomes. In addition, diagnostic methods for identification of SC were also investigated. To date, this study is the largest salivary gland SC cohort reported in Canada.

## Methods

### Patient selection and study design

All patients currently diagnosed with SC in the Canadian Maritime provinces were included in this study, including those with an alternative original diagnosis. Patients were identified using an institutional pathology database at the Queen Elizabeth II Health Science Center in Halifax, Nova Scotia; as well as from the consultation files of MJB. There were no exclusion criteria overall for the study. Particular patients were excluded if chart information was lacking, and are identified appropriately in the results section.

Retrospective chart review was carried out on all patients and included review of initial consultations, follow up reports, operative reports, and pathology review.

### Diagnosis

Diagnosis was made by immunohistochemistry and histopathology with or without confirmation of the diagnostic ETV6-NTRK3 fusion gene via fluorescence in situ hybridization (FISH). Confirmatory FISH was performed at the Molecular Diagnostics Laboratory, University of Nebraska Medical Center.

Staging was by the American Joint Committee on Cancer Seventh Edition.

### Statistics and research approval

Statistical analysis was completed using the commercially available software SPSS (v21; IBM, Chicago, Illinois). Overall survival and locoregional control rates were determined using Kaplan-Meier curves. Overall survival was calculated with events being considered any cause of patient death, with patients alive at time of last follow-up being censored. Local and locoregional control rates were calculated with events being considered either local or regional recurrences, and patients with no previous recurrence at time of last follow-up, or at time of death, being censored.

The Nova Scotia Research Ethics Board and Quality Improvement & Patient Safety Committee has approved this study as a Quality Assurance/Delivery of Care Initiative under Article 2.5 of the Tri-Council Policy Statement 2.

### Literature review

Literature review was carried out in a non-systematic manner. Both MEDLINE/PubMed and Google Scholar searches were completed with the following search terms: “mammary analogue secretory carcinoma,” and “secretory carcinoma AND salivary gland.” English language articles between 2010 and 2018 were included. Citation lists of included articles were also reviewed for possible missing references. Articles were excluded only if they did not identify patient outcomes.

## Results

### Clinicopathological features

In total, 13 patients were identified. Patient demographics are presented in Table 1. There was no gender predominance and patients were young overall (mean age 54 years). The majority of patients were considered smokers. This series includes the youngest described patient in the current literature, a 6-year-old male with a parotid tumor.

**Table 1:** Patient demographics and TNM Staging

Variable	Value
Male Gender	7 (54)
Age	54 (6–84)
Smoker	6 (54)^a^
TNM	
T1	6
T2	3
T3	3
T4	1
N0	11
N1	0
N2a	0
N2b	2
N2c	0
N3	0
M0	13
M1	0
Subsites	
Parotid	9
Submandibular	1
Minor Salivary	0
Hard Palate	2
Lip	1

^a^11 patients had known smoking history

Gender = number of patients (%), Age = median year (range years), Smoker = number of patients (%), TNM = number of patients

Staging is presented in Table 1. Tumors were considered small in the majority of cases (T1 or T2), with the majority of patients having no nodal involvement. In those with nodal involvement, both were N2b due to the involvement of multiple ipsilateral lymph nodes. The majority of tumors were parotid in origin. Minor salivary gland subsites included hard palate and lower lip (Table 1).

### Cytology and pathology

Cytological investigations and their results are presented in Table 2. The majority of patients had a fine needle aspirate performed, none of which yielded a diagnosis of SC. Warthin’s tumor and AciCC were the most common diagnoses on FNA.

**Table 2:** Fine Needle Aspirate results

Patient	FNA Result
001	Benign appearing with appearance of oncocytes and lymphocytes suggestive of Warthin’s Tumor
002	Few groups of epithelioid cells in a background of lymphocytes suggestive of Warthin’s Tumor
003	No FNA performed
004	Mildly atypical cells arranged singly and in sheets with abundant, vacuolated, finely granular cytoplasm suggestive of acinic cell carcinoma vs oncocytic neoplasm
005	Suspicious for malignancy
006	No FNA performed
007	Negative for malignancy
008	No FNA performed
009	No FNA performed
010	Sheets of atypical cells with focally glandular spaces, suggestive of adenocarcinoma vs acinic cell carcinoma vs salivary duct carcinoma
011	Abnormal appearance, suggestive of papillary cystadenoma vs intraductal papilloma, cannot exclude low grade mucoepidermoid
012	Equivocal S100 staining, positive for vimentin and CK7; negative for TTF-1, thyroglobulin, and CD10; suggestive of adenocarcinoma
013	Few malignant cells, positive for AE1 and AE3; negative for LCA and S100; suggestive of poorly differentiated adenocarcinoma

Two patients were originally diagnosed as AciCC, and one was originally diagnosed as adenocarcinoma NOS on initial pathology. Both of these patients were diagnosed prior to the recognition of SC as a distinct tumor entity in 2010. The remaining patients were identified as SC. Three patients were diagnosed on the basis of immunohistochemistry and morphology alone, while the remainder of diagnoses were confirmed with FISH for the ETV6-NTRK3 rearrangement. In the two patients without FISH testing, tumors exhibited characteristic morphological features of SC and were therefore not tested. One patient had negative FISH testing but morphological and IHC features were strongly indicative of SC.

**Fig. 1:**
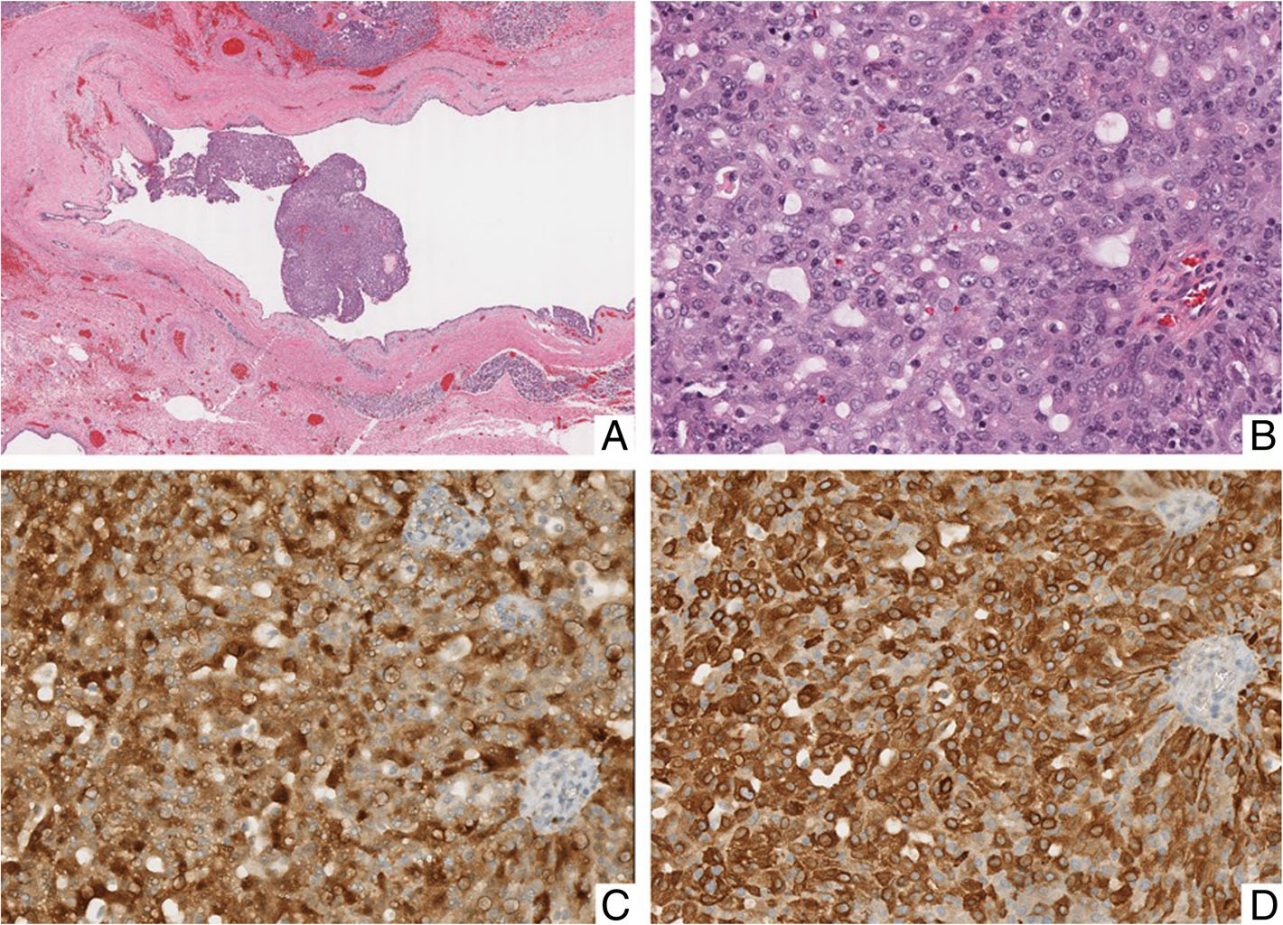
Low power view of the cystic tumor (**a**). High power view demonstrating microcystic and solid architecture (**b**). The tumor cells exhibit strong nuclear staining for S100 (**c**). The tumor cells demonstrate cytoplasmic expression of mammaglobin (**d**)

Three patients displayed perineural invasion on pathology, and one patient displayed lymphovascular invasion. All patients had immunohistochemistry reporting available, of which all were positive for S100 staining. All patients had at least one additional confirmatory stain that was positive beyond S100, with 11 patients having two additional stains. Additional confirmatory stains included vimentin, mammaglobin, or CK7. Figure 1 displays representative histology and immunohistochemistry patterns obtained from the six-year-old patient. Table 3 lists staining patterns.

**Table 3:** Immunohistochemistry staining patterns by patient

Patient	S100	Vimentin	Mammaglobin	CK7
001	+	+	?	+
002	+	+	?	+
003	+	+	?	+
004	+	+	?	+
005	+	+	+	+
006	+	+	+	+
007	+	+	+	+
008	+	?	+	?
009	+	+	+	+
010	+	?	+	?
011	+	+	?	?
012	+	+	?	+
013	+	+	?	+

?: stain not used or not reported

### Morbidity and mortality

Treatment details, complications, and mortality information is presented alongside patient details in Table 4. All patients were treated with primary surgery, with a minority receiving adjuvant radiotherapy. All nine patients with SC originating in the parotid gland received a parotidectomy, seven of which received a neck dissection. One patient with a minor salivary gland tumor underwent extensive resection and free flap reconstruction.

**Table 4:** Clinical history, treatment, and complications

Patient	Clinical History	Treatment	Complications
001	84 years old; Not recorded	Submandibular gland excision	Right marginal mandibular nerve weakness
002	72 years old; Two to three months of gradually increasing parotid mass	Parotidectomy	Transient facial nerve weakness
003	54 years old; Ulcerative lesion posterior to last molar on right maxilla with large odontogenic cyst	Partial maxillectomy, partial soft palate excision, selective neck dissection (Levels II-III), radial forearm free flap, split thickness skin graftAdjuvant radiotherapy (60Gy in 30 fractions)	Minor radiation toxicities
004	55 years old; Ten year history of firm, superficial, round mass in the preauricular area on the left side, increasing in size, with mild ternderness on jaw clench	Superficial parotidectomy, selective neck dissection (level IIA), sternocleidomastoid rotational flap	Mild xerostomia
005	44 years old; Parotid mass present for more than one year	Superficial parotidectomyAdjuvant radiotherapy	None
006	65 years old; Six month history of subcutaneous parotid nodule, stable in size and nontender	Excision of mass, followed by:Superficial parotidectomy, selective neck dissection (Level II)	None
007	6 years old; Fourth month history of stable preauricular mass	Superficial right parotidectomy, selective neck dissection (Level IIA)Adjuvant radiotherapy (60 Gy)Left superficial parotidectomy	Radiation dermatitis
008	43 years old; Slowly growing lesion on lower lip mucosa with central ulceration, present for over one year	Excisional biopsy followed by wide local excision	None
009	27 years old; Slowly growing papillomatous lesion of the hard palate for five years	Local excision	None
010	78 years old; Six month history of slowly growing parotid mass with intermittent sharp, stabbing pain	Subtotal parotidectomy, selective neck dissection (Level II to IV)	None
011	22 years old; Two year history of fluctuating parotid lump	Superficial parotidectomy	None
012	72 years old; Three month history of parotid tail or high cervical mass	Parotidectomy, selective neck dissectionAdjuvant radiotherapy (56 Gy in 33 fractions)	Pain, lymphedema
013	48 years old; Parapharyngeal space involvement, including pterygoid muscles	Transmandibular resection of parapharyngeal mass, subtotal parotidectomy, selective neck dissectionAdjuvant radiotherapy (70 Gy in 35 fractions)	Sacrifice of glossopharyngeal nerve, orocutaneous fistula, eustachian tube scarring requiring tympanostomy, esophageal stenosis requiring dilatation, velopharyngeal insufficiency requiring pharyngoplasty with Radiesse, radiation associated toxicities

Complications related to treatment included radiation toxicity, marginal mandibular weakness, velopharyngeal insufficiency requiring surgical correction, and pharyngocutaneous fistula.

The five-year locoregional control rate was 83% (Additional file 1: Figure S1), with one patient having recurrence to the posterior triangle, as well as pulmonary metastasis. The five-year overall survival was 85.7%. There was only one death, in a patient that died secondary to metastatic thyroid cancer unrelated to their diagnosis of SC.

### Case details of metastatic secretory carcinoma

A 48-year-old female presented with a 3.7 × 1.9 cm mass deep to the deep lobe of the parotid gland, with extension into the parapharyngeal space. Fine needle aspirate (FNA) of the lesion revealed malignant cells, but without a specific diagnosis. Surgical excision was carried out in the form of a transmandibular approach to facilitate subtotal parotidectomy, neck dissection, and resection of tumor at the skull base.

The tumor demonstrated a high-grade carcinoma with extensive perineural invasion and positive neck nodes. Original pathological diagnosis was of adenocarcinoma NOS of salivary gland origin. Thus, the patient underwent adjuvant radiotherapy in the form of 60 Gy in 30 fractions to the tumor bed, retropharyngeal lymph nodes, and left neck. An additional 10 Gy over five fractions was administered to the tumor bed and retropharyngeal nodes.

Four years following initial treatment, the patient returned to the otolaryngology clinic with a peri-incisional lesion originally thought to be a traumatic neuroma. Excisional biopsy was performed and revealed malignant cells. Immunohistochemistry was in keeping with her previous parotid cancer, confirming regional metastasis. Secretory carcinoma was suspected and subsequently confirmed by FISH analysis.

During this same time period, surveillance CT revealed a 0.7 cm lung nodule. This lesion would unfortunately expand to 0.9 cm in size. The patient underwent microcoil guided thoracoscopic wedge resection. Pathology of this lesion demonstrated metastatic SC, with negative margins but positive for vascular invasion. Serial surveillance CT scans were chosen in lieu of systemic chemotherapy. The patient has done well following metastasectomy, with no further evidence of recurrence or metastasis, now ten years from her original diagnosis and 5.5 years from her metastasectomy.

### Literature review

Literature review was carried out to investigate patient outcomes following treatment for secretory carcinoma. In total, 37 studies investigating patient outcomes were identified since the original description of secretory carcinoma in 2010 (Table 5) [2, 6–41]. The included studies totaled 227 patients, of which 218 had reliable follow-up for all variables examined in this literature review. Twenty-two studies were case reports or small case series, with only one to four patients. The majority were single patient case reports. The largest study was 36 patients.

**Table 5:** Literature review

Study	Number of Patients	Tumor Location n (%)	Treatment n (%)	Initial Nodal Disease^b^, n (%)	Survival % (cause; timing)	Recurrence n (%); timing	Regional metastasis n (%); timing	Distant metastasis n (%); location timing
Aizawa et al. 2016	1	Parotid: 1 (100)	Surgery: 1 (100)	0 (0)	100%	0 (0)	1 (100); 2 years	0 (0)
Baghai et al. 2017	10	Parotid: 9 (90) MSG: 1 (10)	Surgery: 10 (100) LND: 3 (30) ART: 4 (40) ACRT: 1 (10)	3 (30)	66% (DOD; 24 months, 18 months)	3 (30); 3, 5, 120 months	0	1 (10); bone 15 months
Balanza et al. 2015	1	Parotid: 1 (100)	Surgery: 1 (100)	0 (0)	100%	0 (0)	0 (0)	0 (0)
Boon et al. 2018	31	Parotid: 18 (58) SMG: 1 (3) MSG: 12 (39)	Surgery: 31 (100) LND: 4 (13) ART: 15 (48)	1 (3)	97% (DOC; 48 months)	1 (3); 50 months	0 (0)	0 (0)
Chiosea et al. 2012	36	Parotid: 26 (72) SMG: 3 (10) MSG: 7 (24)	Surgery: 36 (100)LND: 18 (50)ART: 5 (14)ACRT: 2 (6)	4 (11)	97% (DOD; time unknown)	3 (8); time unknown	0 (0)	1 (3); unknown
Cipriani et al. 2017	1	MSG: 1 (100)	Surgery: 1 (100)	0 (0)	0% (DOD; 3 months)	0 (0)	0 (0)	1 (100); lungs 2 months
Cooper et al. 2013	2	MSG: 2 (100)	Surgery: 2 (100)	0 (0)	100%	0 (0)	0 (0)	0 (0)
Din et al. 2016	11	Parotid: 7 (64)SMG: 3 (27)MSG: 1 (9)	Surgery: 10 (91)ART: 2 (18)ACRT: 2 (18)	2 (18)	91% (DOD; 5 years)	3 (38)^a^; time unknown	0 (0)	0 (0)
Drilon et al. 2016	1	Parotid: 1 (100)	Surgery: 1 (100)ART: 1 (100)Revision Surgery: 3 procedureCrizotinibEntrectinib	0 (0)	100%	0 (0)	0 (0)	1 (100); lungs 5.5 years
Fakhoury et al. 2016	1	Parotid: 1 (100)	Surgery: 1 (100)	0 (0)	100%	0 (0)	0 (0)	0 (0)
Griffith et al. 2013	6	Parotid: 4 (67)SMG: 1 (17)MSG: 1 (17)	Surgery: 5 (83)LND: 3 (50)No treatment: 1 (17)	1 (17)	100%	0 (0)	0 (0)	0 (0)
Helkamaa et al. 2015	1	MSG: 1 (100)	Surgery: 1 (100)	0 (0)	100%	0 (0)	0 (0)	0 (0)
Higuchi et al. 2014	7	Parotid: 6 (86)SMG: 1 (14)	Surgery: 7 (100)ART: 1 (14)	0 (0)	100%	0 (0)	0 (0)	0 (0)
Hijazi et al. 2014	1	Parotid: 1 (100)	Surgery: 1 (100)	0 (0)	100%	0 (0)	0 (0)	0 (0)
Hwang et al. 2014	1	Parotid: 1 (100)	Surgery: 1 (100)	0 (0)	100%	0 (0)	0 (0)	0 (0)
Inaba et al. 2015	1	Parotid: 1 (100)	Surgery: 1 (100)	0 (0)	100%	0 (0)	0 (0)	0 (0)
Ito et al. 2015	14	Parotid: 9 (64)SMG: 1 (7)MSG: 4 (29)	Surgery: 14 (100)	2 (14)	100%	1 (20)^a^; 90 months		0 (0)
Jackson et al. 2017	1	Parotid: 1 (100)	Surgery: 1 (100)	0 (0)	100%	0 (0)	0 (0)	0 (0)
Jung et al. 2013	13	Parotid: 11 (85)Unknown: 2 (15)	Surgery: 13 (100)ART: 2 (15)	0 (0)	100%	3 (23); 10–101 months (mean 44)	0 (0)	0 (0)
Jung et al. 2015	9	Parotid: 9 (100)	Unknown	Unknown	Unknown	3 (33); time unknown	0 (0)	0 (0)
Kratochvil et al. 2012	2	MSG: 2 (100)	Surgery: 2 (100)	0 (0)	100%	0 (0)	0 (0)	0 (0)
Laco et al. 2013	2	Parotid: 1 (50)SMG: 1 (50)	Surgery: 2 (100)	0 (0)	100%	0 (0)	0 (0)	0 (0)
Levine et al. 2014	1	Parotid: 1 (100)	Surgery: 1 (100)	0 (0)	100%	0 (0)	0 (0)	0 (0)
Luk et al. 2015	9	Parotid: 9 (100)	Surgery: 9 (100)LND: 1 (11)ART: 1 (11)	1 (11)	89% (DOD; 13 months)	0 (0)	1 (11); 12 months	0 (0)
Luo et al. 2014	1	MSG: 1 (100)	Surgery: 1 (100)ART: 1 (100)	1 (100)	100%	0 (0)	0 (0)	0 (0)
Majewska et al. 2015	7	Parotid: 6 (86)MSG: 1 (14)	Surgery: 7 (100)LND: 2 (29)ART: 2 (29)	3 (43)	71% (DOD; 20 months, 79 months)	2 (29); 4 months, 10 months	1 (14); 48 months	0 (0)
Mossinelli et al. 2018	1	Parotid: 1 (100)	Surgery: 1 (100)	0 (0)	100%	0 (0)	0 (0)	0 (0)
Ngouajio et al. 2017	1	Parotid: 1 (100)	Surgery: 1 (100)LND: 1 (100)	0 (0)	100%	0 (0)	0 (0)	0 (0)
Oza et al. 2016	3	Parotid: 3 (100)	Surgery: 3 (100)	0 (0)	100%	0 (0)	0 (0)	0 (0)
Rastatter et al. 2012	1	Parotid: 1 (100)	Surgery: 1 (100)LND: 1 (100)	1 (100)	100%	0 (0)	0 (0)	0 (0)
Salat et al. 2015	2	Parotid: 2 (100)	Surgery: 2 (100)LND: 1 (50)ART: 2 (100)	0 (0)	100%	0 (0)	0 (0)	0 (0)
Serrano-Arevalo et al. 2015	4	Parotid: 1 (25)SMG: 1 (25)MSG: 2 (50)	Surgery: 2 (50)Nothing: 2 (50)ART: 1 (25)	1(25)	100%	0 (0)	0 (0)	0 (0)
Shah et al. 2015	1	Parotid: 1 (100)	Surgery: 1 (100)	0 (0)	100%	0 (0)	0 (0)	0 (0)
Skalova et al. 2010	16	Parotid: 13 (81)MSG: 3 (19)	Surgery: 16 (100)ART: 7 (44)LND: 1 (6)	1 (6)	94% (DOD; 6 years)	3 (19), 6 months, 2 years, 6 years	1 (6); 86 months	1 (6); lungs 2 years
Skalova et al. 2014	3	Parotid: 3 (100)	Surgery: 3 (100)ART: 2 (67)LND: 1 (33)	0 (0)	0% (3 DOD; 20 months, 4 years, 6 years)	2 (66); 2 years, 6 years	2 (67); 20 months, 4 years	2 (67); dissemintated 20 months, 4 years
Skalova et al. 2018	10	Parotid: 7 (70)SMG: 2 (20)MSG: 1 (10)	Surgery: 9 (90)LND: 1 (10)ART: 1 (10)ACRT: 1 (10)Nothing: 1 (10)	1 (10)	78% (1 DOD; 2 years. 1 DOC; 3 years)^a^	0 (0)	1 (11); time unknown	1 (11); bone 15 months
Stevens et al. 2015	14	Parotid: 9 (64)Thyroid: 1 (7)SMG: 1 (7)MSG: 3 (21)	Surgery: 12 (88)Nothing: 2 (14)ART: 3 (21)	2 (14)	100%	1 (7); 4 years	0 (0)	1 (7);lungs 4 years
Total	227	Parotid: 166 (73)Other: 61 (27)	Surgery: 211 (97)^c^LND: 37 (17)ART: 56 (26)	24 (11)	93% (13 DOD, 2 DOC)^c^Mean time: 38 months^d^	26 (12)	8 (4)	9 (4)Mean time: 30 months^c^, ^d^

*ART* adjuvant radiotherapy, *ACRT* adjuvant chemoradiotherapy, *LND* lymph node dissection, *MSG* minor salivary gland, *SMG* submandibular gland, *DOD* died of disease, *DOC* died of other causes

^a^study includes patients lost to follow up, ^b^: category includes patients with clinically evident nodes, or those that had lymph node dissection, ^c^: Jung et al. 2014 not included, ^d^: Mean time dos not include Chiosea et al

The most common primary tumor site was the parotid gland (73%). Of the remaining patients, 43 (19%) primarily involved the minor salivary glands, and 15 involved the submandibular gland (7%). Initial nodal disease was uncommon, with only 11% of patients presenting with clinically evident lymph nodes or positive nodes after lymph node dissection.

The majority of patients underwent surgical resection (97%), which included a combination of wide local excision, superficial and partial parotidectomy, total parotidectomy and radical parotidectomy. Lymph node dissection was relatively uncommon, as was adjuvant therapy. In total, 17% of patients underwent lymph node dissection of some form, while 26% of patients received adjuvant radiotherapy. Interestingly, one case report examined the possibility of tyrosine kinase inhibitors for the treatment of secretory carcinoma. Several patients did not undergo treatment.

Outcomes were generally favorable. Overall locoregional recurrence was 16%, which included both local recurrence, as well as lymph node metastasis. Distant metastasis was uncommon, with only nine describe reports (4%). In those patients with information available for analysis, mean time to distant metastasis development was 30 months. Four patients with distant metastasis had lung or pleural involvement, two had bone involvement, and two had disseminated disease. The overall survival was 93%, with mean time to death of 38 months after initial treatment. Of those deaths described, 87% were directly due to disease progression.

In those patients that died due to disease, five died due to distant metastasis, with the remainder dying as a result of aggressive locoregional recurrence, including several instances of temporal bone invasion. Skalova’s group, in three separate studies, described the greatest number of deaths, including one paper focused on high grade transformation, and another series that includes one patient demonstrating high grade transformation. All of these patients died due to their disease. Excluding patients with high grade transformation, only nine patients died due to disease, giving a disease-specific survival of 98%.

## Discussion

Secretory carcinoma is a recently described malignancy affecting the salivary glands of the head and neck. It expresses the translocation mutation t (12;15) (p13;q25), which results in the fusion gene ETV6-NTRK3 [2], a characteristic it shares with breast carcinoma of the same name. While several other chromosomal translocations have recently been described in salivary gland tumors, such as EWSR1-ATF1 in hyalinizing clear cell carcinoma and PRKD1–3 gene translocations in cribiform adenocarcinomas of the minor salivary glands [39], there remains a paucity of data surrounding the natural history, diagnosis, treatment, and outcomes of this relatively rare disease.

The initial presentation of SC seems to be mostly uniform. The majority of patients in our series presented with slowly growing, painless masses of the parotid, neck or oral cavity. The minority presented with more rapid growth, aggressive involvement of deep structures, or pain. This is similar to the largest series to date, presented by Chiosea and colleagues, in which 94% of patients presented with a painless mass [6]. Despite a relatively uniform presentation, the age of presentation varied substantially, with the youngest patient being only six years of age. This is amongst the youngest patient described to date [21]. The extremity of this patients age made treatment decisions initially difficult, given there was no data to guide these choices. As with other studies, the most common subsite for presentation in our study was the parotid gland, followed by minor salivary glands, and finally the submandibular gland was involved in a single patient. However, as evidenced by the metastatic SC in this case series, there is potential for aggressive presentations of this disease.

Interestingly, 75% of patients were originally diagnosed with SC in our study. In the three patients initially diagnosed as AciCC or adenocarcinoma NOS, each was diagnosed prior to the initial description of SC. Unfortunately, FNA was not particularly helpful in diagnosis of SC, with no patients receiving this diagnosis by FNA alone. Only 4 of 9 aspirates were correctly diagnosed as either malignant or suspicious for malignancy. The most common diagnoses by FNA were Warthin tumor and AciCC. Generally, the accuracy of fine needle aspiration for salivary gland tumors is good. However, certain diagnoses are relatively easy to make (e.g. benign mixed tumor, Warthin tumor, and high grade malignancies) while other diagnoses are difficult because many salivary malignancies (including SC) have low grade cytological features and there is overlap between benign and malignant. The cytopathology community has been working to address these issues by developing a classification similar to the Besthesda thyroid classification, in which salivary gland cytopathology specimens are stratified into diagnostic categories with inherent rates of malignancy (Rossi et al. 2018). The cytological features of SC are not well known due to its only recent description (especially when diagnoses in this study were made) and its still unfamiliarity to most cytopathologists. However, the ability to prospectively diagnosis SC with cytology has been previously described [16]. In the study by Griffith and colleagues, SC was found to form papillary groups on cytology, with abundant, prominent or multivacuolated cytoplasm. Both AciCC and SC have similar cellular arrangement on cytology smears. However, SC tends to have increased extracellular and intracellular mucin compared to AciCC, and a greater variation in the size of the cytoplasmic vacuoles found.

Of those patients with FISH data available, there was a high correlation between positivity for an ETV6 rearrangement and varying combinations of S100, vimentin, and mammaglobulin staining. One study demonstrated a 95% correlation between combined morphologic and immunohistochemical features of SC, and the presence of the defining rearrangement [38]. Eleven patients in our series underwent testing with FISH, with ten showing the characteristic translocation. One case showed no evidence of an ETV6 rearrangement, but morphologically and immunohistochemically it was a classic SC. The case was referred for an outside pathology consultation and the consultant agreed with the diagnosis of SC. It is now apparent that in some cases of SC, alternative gene rearrangements are possible, such as ETV6 rearrangements with yet discovered partners (ETV6-X), atypical fusion junctions with NTRK [42], as well as ETV6-RET fusion [43]. All patients in our study had S100 immunohistochemistry available, and all were positive. Additionally, all patients had at least one other positive confirmatory stain. Two other cases were not sent for FISH testing but were consistent by IHC and morphology as described below.

In all cases, the IHC results and morphological features were consistent with the diagnosis of SC (see below), although the staining panels used differed slightly from case to case, largely because they were diagnosed at different times while knowledge of the IHC patterns evolved. Some cases were given a different diagnosis initially because the SC entity was newly described and not widely known. There are many salivary gland tumors with characteristic translocations in which proving the presence of a translocation is not required for the diagnosis (e.g. adenoid cystic carcinoma). The same is *now* true of SC because we are becoming more familiar with the typical morphology and IHC. Basically, at our institution, we would require: typical morphology (as described below), positivity for S100 and another confirmatory stain, and lack of PAS-positive zymogen granules (to exclude acinic cell ca). Confirmatory FISH is currently preferred, but not required by some expert pathologists, especially in cases with typical features.

Characteristic morphological features of SC, include lobulated and/or cystic architecture with tumor cells having fairly abundant, eosinophilic vacuolated cytoplasm, bubbly secretory material, mucin production and low grade vesicular nuclei. Immunohistochemical staining patterns consistent with SC include strong S100 positivity, as well as CK7, vimentin, mammaglobin, and GATA3. They should not be positive for basal/myoepithelial markers, such as p63, nor should they display significant numbers of PAS-positive zymogen granules, which excludes acinic cell carcinoma.

Oncological characteristics and outcomes were similar to previous studies. Tumors were more advanced in our study compared to Chiosea et al., in which 81% were T1 or T2, compared to only 69% in our study. Two had positive lymph nodes (15%). Our patients tended to have more advanced nodal disease (15% N2b, versus 6%). This rate of regional lymph node involvement is overall slightly less than that reported by Chiosea and colleagues, but remains higher than the rate found in ACiCC [6, 44]. Only one patient in our series was found to have locoregional recurrence, and this patient also had distant metastasis (7.7%). This rate of recurrence is similar to Chiosea and colleagues, while the rate of metastasis is higher in our series. Although the 5-year control rate is not reported in Chiosea et al’s study, extrapolating from their Kaplan-Meier curve shows eight of 28 patients recurred, five of which were within the first five years (18% recurrence rate before five years). In a study by Jung and colleagues, three of nine patients had recurrence of disease, with a median time to recurrence of 44 months. The only death in our series was unrelated to the diagnosis of SC. It is recognized that both our case series and that by Chiosea and colleagues have small sample sizes and smaller still event rates.

Literature surrounding treatment and outcomes for SC of the salivary glands is somewhat limited. Only 218 patients, over 36 studies, could be reliably included in all variables examined in our literature review, and several key factors could not accurately be examined. Notably, TNM staging, extent of surgical resection, pathology results, radiotherapy intensity, and chemotherapy details were not examined and would offer key pieces of information in deciding future treatment. As well, given that the majority of included patients were identified retrospectively, information applicable to treatment decision making is limited. Yet another limitation is length of followup, with the majority of studies being well below five years for most patients. Of course, this is owing to SC only recently being described. However, it seems clear that aside from patients with high grade transformation, long term survival is favorable.

The vast majority of SC are considered to be histologically or cytologically “low grade”, even when the correct diagnosis is not made initially. This is evident in the fact that many fine needle aspirates are diagnosed as benign lesions, such a Warthin’s tumor. In one case in our series, the patient with lung metastases initially was diagnosed as “high grade adenocarcinoma-NOS” and only recognized as SC once it had recurred (and following the initial recognition of the entity). This case was chosen to highlight the possible aggressive nature that SC may display, and documents one of the few cases of distant metastasis in SC, including one of the only patients to survive. The diagnosis of a high-grade adenocarcinoma was based on increased nuclear atypia and mitotic activity. Thus, there is a spectrum of atypia in SC, from morphologically bland to those tumors with more obvious malignant features.

Although the above case was not considered to be an example, many salivary gland carcinomas, such as acinic cell carcinoma, adenoid cystic carcinoma and epithelial-myoepithelial carcinoma, can undergo “high grade transformation” in which the tumor is transformed to a high-grade malignancy that is different from the original tumor [45]. Secretory carcinoma with high-grade transformation resulting in recurrence, metastasis, and cancer related death has been previously described in three cases by Skalova and colleagues [40]. In their report, the first descriptions of high-grade SC morphology were provided. In all three cases, the patients presented with recurrence and died within two to six years following initial treatment.

As has been previously suggested elsewhere, patients in this case series were managed similar to AciCC [3]. All patients underwent primary surgery. Four patients were offered adjuvant radiotherapy. Complications were primarily related to radiation toxicity, and included esophageal stenosis, skin reactions, mucositis, and dysphagia. However, patients did also experience complications related to surgery, including prolonged marginal mandibular nerve weakness, pharyngocutaneous fistulas, and velopharyngeal insufficiency requiring operative intervention.

Further investigations should be completed across multiple institutions in order to bolster the population sizes examined. Despite being more common than originally suspected, SC remains rare overall. In order to accrue patients required to elucidate optimal management, multiple institutions would likely be required.

## Conclusion

In summary, SC is a recently described disease entity affecting the head and neck salivary glands. Diagnosis may occur through typical immunohistochemistry and morphological patterns, and should be confirmed in most cases by detection of the ETV6-NTRK3 fusion gene. Patients generally fair well, although the rates of regional lymph node involvement are higher than AciCC. The currently presented series is the largest set of Canadian patients, the only to focus purely on Canadian patients, and amongst the largest series overall. As well, we provide the most comprehensive literature review to date, with a focus on treatment and outcomes for this disease entity.

## Additional file

Additional file 1: **Figure S1.** Kaplan-Meier curve representing locoregional control rates. (TIFF 14826 kb)

## Abbreviations

AciCC: Acinic Cell Carcinoma
FISH: Fluorescence in situ hybridization
FNA: Fine Needle Aspiration
NOS: Not Otherwise Specified
SC: Secretory Carcinoma
