# Supplementary figures and images for: Secretory carcinoma: the eastern Canadian experience and literature review
Source: J Otolaryngol Head Neck Surg. 2018 Nov 16;47:69. doi: 10.1186/s40463-018-0315-6 (PMC6240209; doi:10.1186/s40463-018-0315-6)

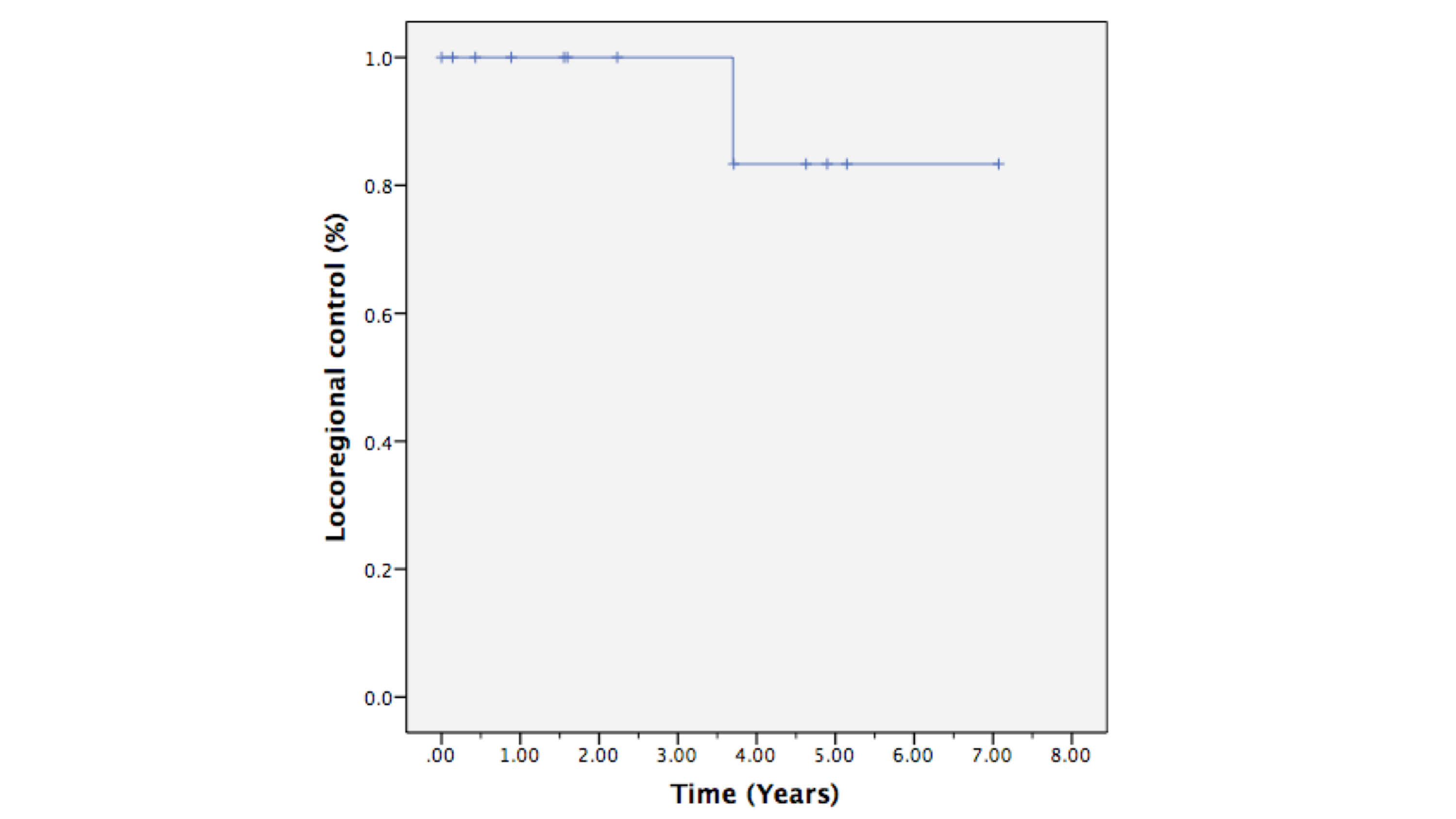

Supplement: Supplementary file 1 — Figure S1. Kaplan-Meier curve representing locoregional control rates. (TIFF 14826 kb) [file 40463_2018_315_MOESM1_ESM.tiff]
